# The therapeutic potential of bacteriophages targeting gram-negative bacteria using *Galleria mellonella* infection model

**DOI:** 10.1186/s12866-018-1234-4

**Published:** 2018-08-31

**Authors:** Prasanth Manohar, Ramesh Nachimuthu, Bruno S. Lopes

**Affiliations:** 1 Antibiotic Resistance and Phage Therapy Laboratory, School of Bio Sciences and Technology, Vellore Institute of Technology (VIT), Vellore, Tamil Nadu 632014 India; 2 School of Medicine, Medical Sciences and Nutrition, Medical Microbiology, University of Aberdeen, Aberdeen, AB25 2ZD UK

**Keywords:** *G. Mellonella*, Phage therapy, *Escherichia coli*, *Klebsiella pneumoniae*, *Enterobacter cloacae*

## Abstract

**Background:**

Phage therapy is the therapeutic use of bacteriophages to treat highly drug resistant bacterial infections. The current surge in bacteriophage therapy is motivated mainly because of the emergence of antibiotic-resistant bacteria in clinics. This study evaluated the therapeutic potential of three bacteriophages isolated against *Escherichia coli* ec311, *Klebsiella pneumoniae* kp235 and *Enterobacter cloacae* el140 strains using *Galleria mellonella*. The in vitro activity of three different phages belonging to *Podoviridae* and *Myoviridae* families was studied by the double agar overlay method against multi-drug resistant strains. Larval survivability studies were performed to evaluate the potential of phages against infection using *G. mellonella*.

**Results:**

All the three phages were found to have potential to infect the host bacterial strains. For in vivo studies it was observed that *E. coli* and *E. cloacae* infected larvae, should be treated with three phage doses (20 μL, 10^4^ PFU/mL) at 6 h interval to achieve 100% survival rate. But in the case of *K. pneumoniae*, a single phage dose treatment showed promising outcome. When mixed bacterial infections (all three bacterial cultures at 10^8^ CFU/mL) were tested, minimum of four doses of phage cocktail (three phages) at 6 h interval was necessary to recover the larvae. All the results were confirmed by enumerating bacteria from the larvae.

**Conclusion:**

Our data shows that although in vitro studies showed high infectivity of phages, for in vivo models multiple phage doses were required for effective treatment.

**Electronic supplementary material:**

The online version of this article (10.1186/s12866-018-1234-4) contains supplementary material, which is available to authorized users.

## Background

Antibiotics are considered as a “miracle drugs” to treat bacterial infections but due to the cheaper availability of antibiotics, the use of antibiotics has become common and widespread throughout the world [1]. The irrational and uncontrolled use of antibiotics leads to bacterial resistance, which is of major concern to combat serious life threatening bacterial infections [1, 2]. *Escherichia coli*, *Klebsiella pneumoniae* and *Enterobacter cloacae* are Gram-negative bacilli which fall in the ESKAPE (*Enterococcus faecium*, *Staphylococcus aureus*, *Klebsiella pneumoniae*, *Acinetobacter baumannii*, *Pseudomonas aeruginosa*, and *Enterobacter* species) group of pathogens and cause serious community or hospital acquired infections [3]. Bacteria becoming resistant to last resort of antibiotics such as carbapenems and colistin make clinical treatment options for infections very limited [4].

Multi-drug resistance among *Enterobacteriaceae* is growing at a faster rate than once believed, which results in high mortality rate globally [4]. In order to overcome this problem of resistance a more effective alternative therapeutic option is required. As bacteriophages are very good bio-agents which feed on bacteria, they are considered as a potential alternative to control the bacterial multiplication in the environment as well as in infections [5, 6]. Phage therapy involves the use of bacteriophages for the treatment of bacterial infections and has gained a renewed interest because of antibiotic resistance in bacteria [7–9]. For in vivo studies, murine models are one of the commonly used animal models for studying bacterial infections and phage therapy [10]. *G. mellonella*, a larger wax moth larva has been found to be a useful model to study the pathogenesis of bacterial infections and to study the efficacy of antibacterial drugs [11]. Additionally, *G. mellonella* is an ideal model because they do not require ethical approval, have shorter life span, can be grown at 37 °C and they have similarities to vertebrates innate immune responses [12]. In earlier studies, *G. mellonella* has been successfully used to evaluate the effectiveness of experimental phage therapy [10]. In this study, we examined the in vivo therapeutic efficiency of three bacteriophages; *Escherichia* phage ECP311 (ECP311), *Klebsiella* phage KPP235 (KPP235) and *Enterobacter* phage ELP140 (ELP140) against *E. coli*, *K. pneumoniae* and *E. cloacae* strains, using *G. mellonella* as a model organism. We investigated the effective dosage of target phages required to reduce the lethality of bacterial infections in *G. mellonella* model and evaluated the dose dependent potential of phage cocktail (three phages each at 10^4^ PFU/mL) to target multiple bacterial infection.

## Results

### Bacteriophage characteristics

The bacteriophages used in this study were characterized for their lytic activity against the target bacteria. Accordingly, ECP311 belonged to *Phieco32likevirus* (*Podoviridae* family) and has the adsorption velocity of 1.1 × 10^− 9^ mL/min, latency period of 26 min and a burst size of 180 phage particles/infected cell, KPP235 belonged to *Podoviridae* of phages with the adsorption velocity of 4.35 × 10^− 9^ mL/min, latent period of 40 min and a burst size of 120 phage particles/infected cell while ELP140 belonged to *Myoviridae* and has an adsorption velocity, latent period and burst size of 2.8 × 10^− 9^ mL/min, 11 min and 135 phages/infected cell (Additional file 1: Figure S1). ECP311 was found to be lytic against 43 *E. coli* isolates that were belonging to different pathotypes, KPP235 was lytic against 17 different strains of *K. pneumoniae* and ELP140 against 11 *E. cloacae* (*n* = 15), 2 *E. hormaechei*, 2 *E. asburiae* and 2 *E. aerogenes* isolates. In this study only the host bacterial multi-drug resistant strains, *E. coli* ec311, *K. pneumoniae* kp235 and *E. cloacae* el140, against which these phages showed maximum lytic activity were used (Additional file 2: Table S1). The stability of these phages were tested at different pH conditions by exposing them to varying range of pH and the results showed that all the three phage were viable from pH 3 to 11 and they are able to show activity from 20 °C to 55 °C (data not shown).

### In vivo efficiency of phage treatment

All three phages (ECP311, KPP235 and ELP140) were used for in vivo studies using *G. mellonella* (wax moth larvae) model. The lethality of all the three bacteria against the larvae was tested and the range 10^4^ to 10^8^ CFU/mL was found to be highly lethal with all larvae dead within 48 h period at 10^8^ CFU/mL (20 μL volume) concentrations. Further experiments were performed using a bacterial concentration of 10^8^ CFU/mL. In the set of uninfected and saline injected larvae used as a control 100% survivability obtained. The prepared phage lysate (10^4^ PFU/mL) was injected (20 μL) in another set of larvae and 100% survival rate was observed that showed the prepared phage lysate for the treatment studies were not lethal to the larvae even at higher doses. All the three above mentioned groups were used as a control during the treatment studies (Table 1). Prophylactic models were studied in which larvae were infected with phages 2 h prior to their infection with bacteria, but no observable differences in survival rates were noted (data not shown).

**Table 1:** Experiment setup to evaluate the efficiency of phage therapy for the treatment of *G. mellonella* using single phage dose, multiple phage doses and phage cocktails

Regimen	Model	Treatment/condition
Control groups		
SET 1	Control I (untreated)	Larvae were injected with 20 μL of saline.
SET 2	Control II	Larvae were infected with 20 μL of bacteria (10^8^ CFU/mL).
SET 3	Control III	Larvae were injected with 20 μL of phage lysate (10^4^ PFU/mL).
Single Phage Dose		
SET 4	Test-1	Larvae were infected with 20 μL of bacteria and treated with 20 μL phages within 1 h.
Multiple Phage Doses		
GROUP-1	Single dose	Larvae were infected with 20 μL of bacteria and treated at 0th hour using 20 μL of phages.
GROUP-2	Double dose	Larvae were infected with 20 μL of bacteria and treated with two doses (20 μL each) of phages at 0th hour and at 6th hour.
GROUP-3	Triple dose	Larvae were infected with 20 μL of bacteria and treated with three doses (20 μL each) of phages at 0th hour, at 6th hour and 12th hours.
GROUP-4	Quadruple dose	Larvae were infected with 20 μL of bacteria and treated with four doses (20 μL each) of phages at 0th hour, at 6th, 12th and 24th hours.
Cocktail of Phages		
GROUP-A	Single cocktail dose	Larvae were infected with 20 μL of bacterial mixture and treated at 0th hour using 20 μL of phage cocktail.
GROUP-B	Double cocktail dose	Larvae were infected with 20 μL of bacterial mixture and treated at 0th hour and at 6th hour using 20 μL of phage cocktail.
GROUP-C	Triple cocktail dose	Larvae were infected with 20 μL of bacterial mixture and treated at 0th hour and at 6th, 12th hour using 20 μL of phage cocktail.
GROUP-D	Quadruple cocktail dose	Larvae were infected with 20 μL of bacterial mixture and treated at 0th hour and at 6th, 12th, 24th hour using 20 μL of phage cocktail.
GROUP-E	Quintuple cocktail dose	Larvae were infected with 20 μL of bacterial mixture and treated at 0th hour and at 6th, 12th, 24th, 30th hour using 20 μL of phage cocktail.

*In each group 10 larvae were used for the study. For all the groups, the survivability of the larvae was evaluated for 96 h and the results were interpreted statistically

**Fig. 1:**
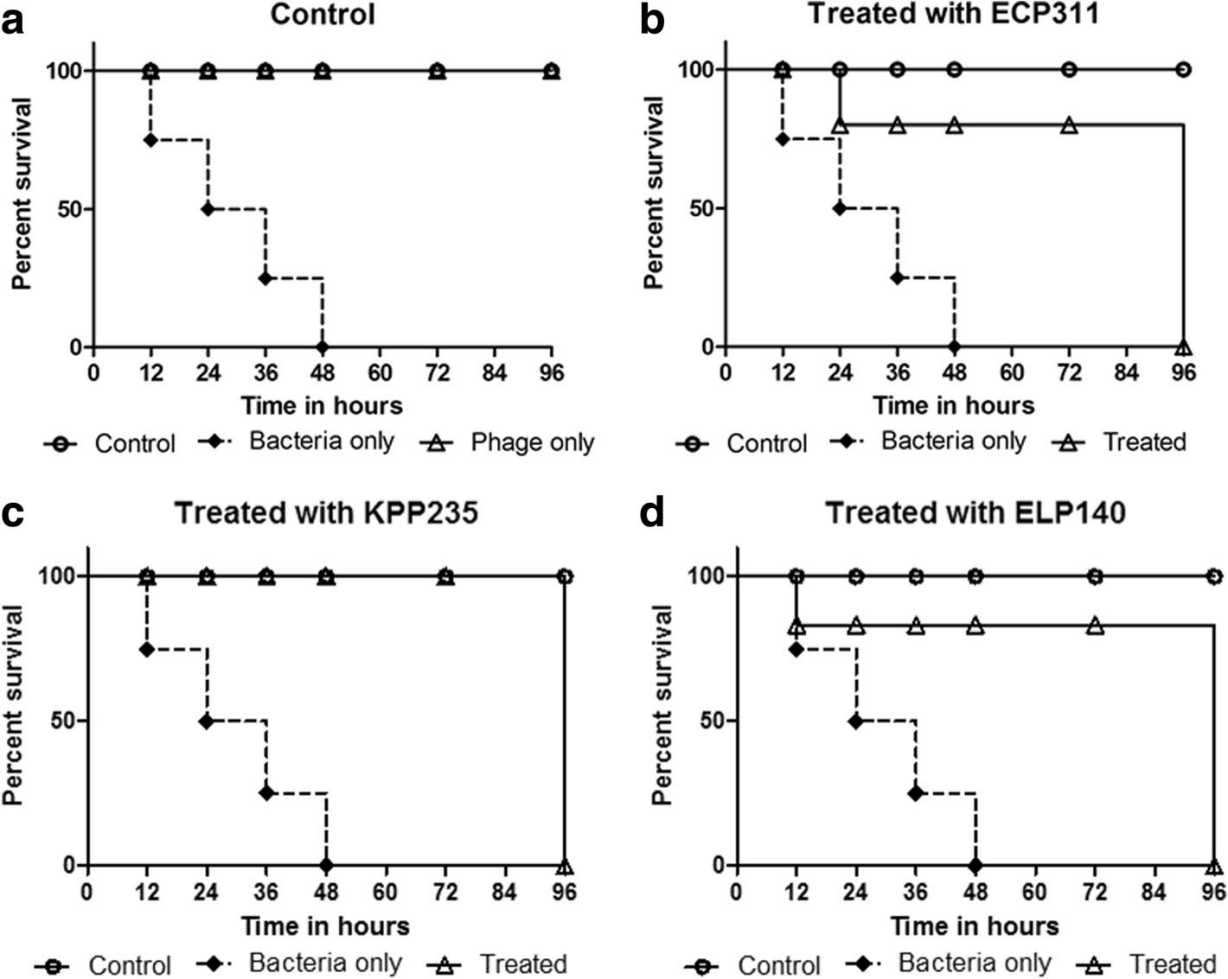
Impact of single dose phage treatment on *E. coli* ec311, *K. pneumoniae* kp235 and *E. cloacae* el140 infection and survival rates of *G. mellonella* larvae. A single phage dose (10^4^ PFU/mL) was injected at 0^th^ hour after the larvae was pre-infected with 10^8^ CFU/mL of bacteria. **a** Saline, phage and bacteria only control group larval survival. **b** Survival rates of the larvae infected with *E. coli* ec311 treated with single dose of *Escherichia* phage ECP311 (*p* = 0.0045). **c** Survival rates of the larvae infected with *K. pneumoniae* kp235 treated with single dose *Klebsiella* phage KPP235 (*p* = 0.0006). **d** Survival rates of larvae infected with *E. cloacae* el140 treated with *Enterobacter* phage ELP140 (*p* = 0.0052). The survival rates were plotted using the Kaplan-Meier method and log-rank test was used to analyze the difference in survival rates in GraphPad Prism 7.0. All phage treatment results were compared with the control. A statistically significant difference (*p* < 0.05) was observed using 10 worms per group on phage treatment

**Fig. 2:**
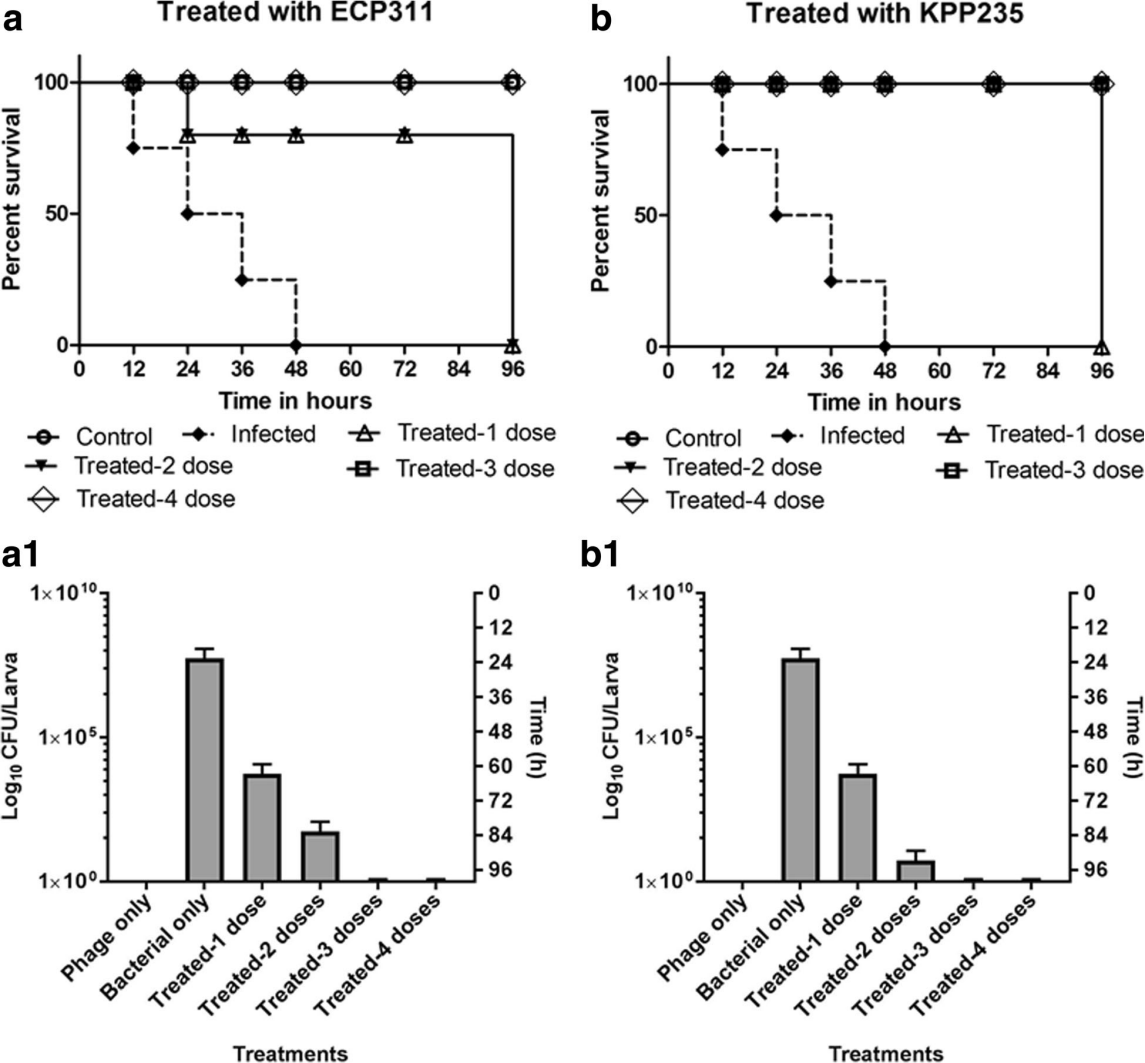
Impact of multiple phage doses on survivability of larvae that was infected with *E. coli* ec311 and *K. pneumoniae* kp235. The multiple phage doses (10^4^ PFU/mL) were injected at 0^th^, 6^th^, 12^th^ and 24th hour after the larvae were pre-infected with 10^8^ CFU/mL of bacteria. All the phage treatment results were compared with the control. **a** Impact of multiple doses of *Escherichia* phage ECP311 on the survivability of larvae infected with *E. coli* ec311 (*p* = 0.0002). **a1** Bacterial enumeration from larvae, results are represented as CFU of bacteria/larva. **b** Impact of multiple doses of *Klebsiella* phage KPP235 on the survivability of larvae infected with *K. pneumoniae* kp235 (*p* = 0.0001). **b1** Bacterial enumerations from larvae, results are represented as CFU of bacteria/larva. The survival rates were plotted using the Kaplan-Meier method and log-rank test was used to analyze the difference in survival rates. A statistically significant difference (*p* < 0.05) was observed using 10 worms per group on phage treatment. Error bars represent standard error of the mean (SEM) of three independent replicates and data were analyzed using GraphPad Prism 7.0

For the single phage dose treatment, the bacterial inoculum (20 μL) of 10^8^ CFU/mL and the phage titer (20 μL) of 10^4^ PFU/mL was used, and phages were injected within 1 h of bacterial infection. The phage treatment results were observed at 12, 24, 36, 48, 72 and 96 h. A single dose of ECP311 was found to be effective for a 12 h period but the survivability of larvae reduced at 24 h, 75% survival rate was observed with all larvae dead within 96 h period (Fig. 1b). This data clearly showed that more than one dose of phages may be required to increase the survivability of larvae that were infected with *E. coli* ec311. To prove this, multiple doses of phages (10^4^ PFU/mL) were given to the infected larvae at 0th, 6th, 12th and 24th hours. The results showed that at the single and double doses of phage treatment (ECP311), the survival rate of larvae were 75% (Fig. 2a) and increased bacterial load in the larvae were also observed (Fig. 2a_1_). At the increased phage doses of three and four, 100% survival rate and complete reduction of bacterial load in the larvae was observed. This clearly showed that in order to treat the larvae that were infected with *E. coli* ec311 at least three doses of phages are required at 6 h interval (Fig. 2a).

During the single dose KPP235 treatment (larvae were infected with *K. pneumoniae* kp235), 100% survivability was observed up to 96 h (Fig. 1c) but lesser movement was noted in larvae after 72 h. Though, the single phage dose treatment showed 100% survival rates, multiple doses of phages were tested further. During the multiple phage dose treatment, 100% survival rate and good larvae movement was observed up to 96 h after two doses of phage treatment at 0^th^h and 6^th^h (Fig. 2b). Further, to determine the bacterial load during the treatment, sacrificed larvae were enumerated to analyze the bacterial count and the data showed that after two phage dose treatments, bacterial load was reduced and after third and four doses there was no bacteria present in the larvae (Fig. 2b_1_). The results showed that even the single dose of phage treatment (KPP235) has a potential effect against the *K. pneumoniae* kp235 infection though the lethality of infection was found to be reduced only after two doses of phage treatment (Fig. 2b, b_1_).

**Fig. 3:**
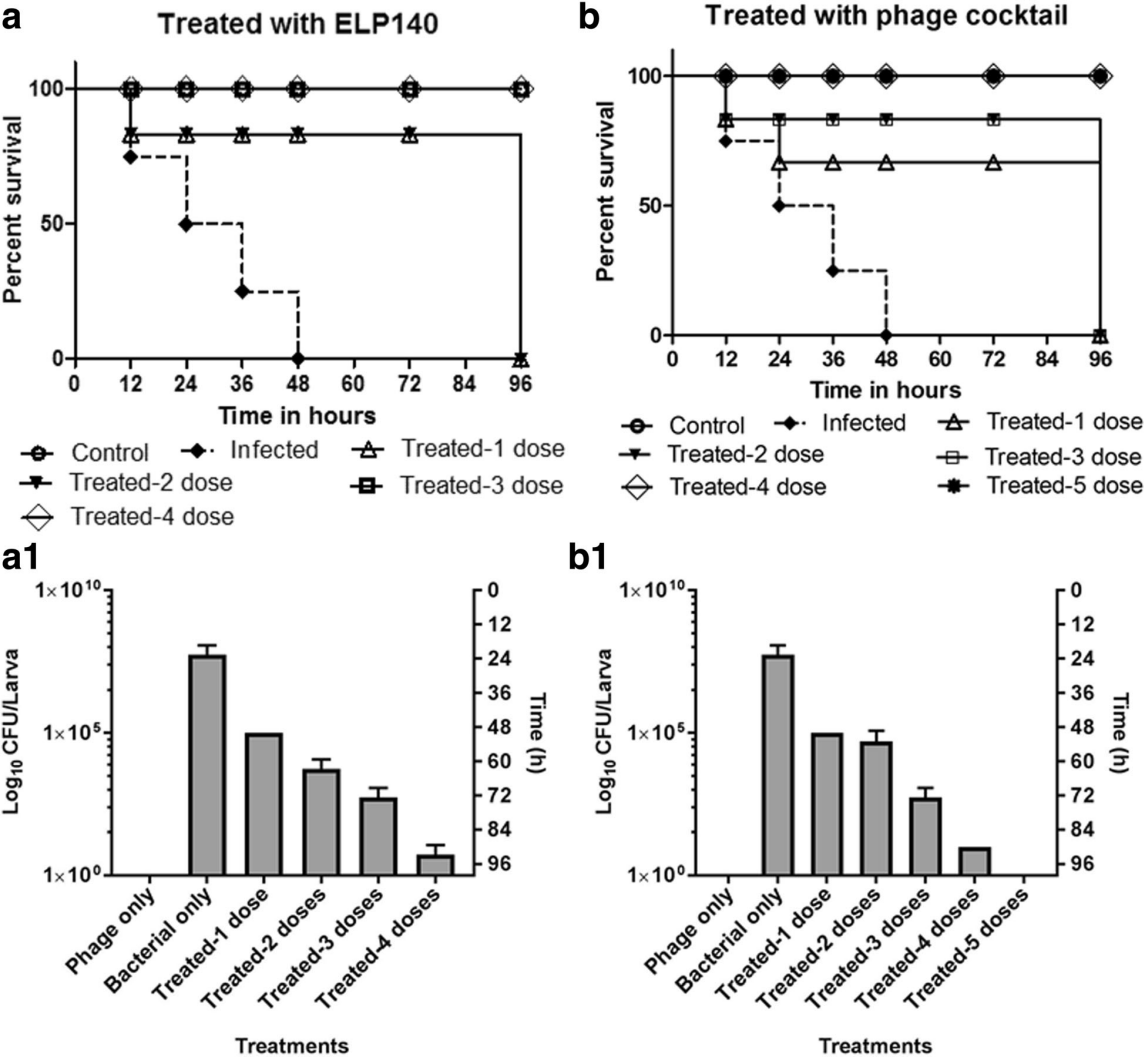
Impact of multiple phage doses on survivability of larvae that was infected with *E. cloacae* el140 and the efficiency of phage cocktail in treating multiple bacterial infections. The multiple doses of phages (10^4^ PFU/mL) were injected at 0^th^, 6^th^, 12^th^, 24^th^ and 30th hour after the larvae was pre-infected with 10^8^ CFU/mL of bacteria. All the phage treatment results were compared with the control. **a** Impact of multiple doses of *Enterobacter* phage ELP140 on the survivability of larvae infected with *E. cloacae* el140 (*p* = 0.0003). **a1** Bacterial enumeration from larvae, results are represented as CFU of bacteria/larva. **b** Phage cocktail studies- Impact of multiple doses of phage cocktail on the survivability of larvae infected with mixed bacteria (*E. coli* ec311, *K. pneumoniae* kp235 and *E. cloacae* el140), for the phage cocktail studies five doses of prepared phage cocktail was used and the results are represented as time to death (*p* = 0.0016). **b1**) Phage cocktail studies- Bacterial enumeration from larvae after the experiment. Results are represented as CFU of bacteria/larva. The survival rates were plotted using the Kaplan-Meier method and log-rank test was used to analyze the difference in survival rates. A statistically significant difference (*p* < 0.05) was observed using 10 worms per group on phage treatment. Error bars represent standard error of the mean (SEM) of three independent replicates and data were analyzed using GraphPad Prism 7.0

The ELP140 application on *E. cloacae* el140 infected larvae showed that a single dose of phage is not sufficient to obtain the 100% survival rate after 96 h. A single dose of ELP140 was effective with a survival rate of 80% and remained constant till 72 h with all larvae dead within 96 h period (Fig. 1d). After three doses of phage treatment at 0th, 6th and 12th, complete reduction of bacterial lethality was noted with 100% survival rate (Fig. 3a). A reduction in bacterial load with the increase in number of phage doses was also observed during the studies using the larval samples (Fig. 3a_1_). In order to increase the larval survivability during the phage treatment (ELP140), at least three phage doses were required to treat the larvae that were infected with *E. cloacae* el140.

To further study the potential of phage therapy against the multiple bacterial infections, another set of studies were performed using the larvae that were infected with mixed bacterial culture (*E. coli* ec311, *K. pneumoniae* kp235 and *E. cloacae* el140, 10^8^ CFU/mL) and treated using the phage cocktail (ECP311, KPP235 and ELP140 at 10^4^ PFU/mL). When single dose of phage cocktail treatment was used, *Enterobacter cloacae* was eliminated completely but *E. coli* and *K. pneumoniae* were present, which led to death of the larvae. To improve the efficiency of phage cocktail treatment, multiple phage doses up to five doses were used. The data showed that until first three doses (0^th^h, 6thh and 12thh), there was only 60% survival rate and there was improvement in larval survival after the fourth (24thh) and fifth (30thh) doses (Fig. 3b). The bacterial load in larval fecal samples was reduced only after four doses of phage (cocktail) treatment (Fig. 3b_1_). This study showed that, for the application of phage cocktail against the larvae infected with *E. coli* ec311, *K. pneumoniae* kp235 and *E. cloacae* el140, a single dose of phage cocktail was found to be less effective but four doses of phage cocktail was very effective and sufficient to produce 100% survival rates in larvae model.

## Discussion

Phages have wide applications and can be used as biocontrol agents in food processing to reduce the bacterial load [13], or to treat bacterial infections in food animals or in eliminating the bacterial pathogens in crops [14–16]. Phage therapy is developing as a promising treatment of choice to cure infections caused by multi-drug resistant bacteria and there are increasing reports on the bacteriophages that are isolated against pathogens such as *E. coli*, *K. pneumoniae* and *E. cloacae* [17–20].

The three phages used in this study, *Escherichia* phage ECP311, *Klebsiella* phage KP235 and *Enterobacter* phage ELP140 showed 100% lytic activity against *E. coli*, *K. pneumoniae* and *E. cloacae* when tested in vitro. Hence, the activity of phages in vivo was examined using *G. mellonella* as a model organism. Initial bacterial virulence studies showed that the test bacterial pathogens (*E. coli*, *K. pneumoniae* and *E. cloacae*) were highly lethal to the larvae and were dead within 48 h at the bacterial load of 10^8^ CFU/mL. A single phage dose (ECP311, KPP235 and ELP140) was found to increase the survival rate of larvae that were infected with bacteria. In the course of the experiment, when single phage dose was used for the treatment, there was a gradual decrease in phage count which subsequently led to the recovery and multiplication of bacteria causing death of the larvae. A similar result was observed by Olszak et al. when PB1-like phage was injected to treat larvae that were infected with *P. aeruginosa* [21]. When multiple phage doses were used to treat larvae infection, three doses of phage injection at 0^th^h, 6thh and 12thh was found to be sufficient to obtain 100% survival rate and complete reduction of bacterial load in larvae was observed in this study. Dosage dependent survival rates was observed in the larvae which showed that even though phages are considered to have auto-dosing, there is a need for minimal dosage to remove the pathogen. In this study, both *Escherichia* phage ECP311 and *Enterobacter* phage ELP140 were found to be less effective during the single phage dose treatment of infected larvae but the *Klebsiella* phage KPP235 was found to increase the survival rate even at single phage dose. Earlier studies showed the importance of phage size and phage generation time that can influence the therapeutic results of phage therapy [21]. The three phages used in this study *Escherichia* phage ECP311; *Klebsiella* phage KPP235 and *Enterobacter* phage ELP140 belonged to *Podoviridae* and *Myoviridae* families respectively. The *Klebsiella* phage KPP235 which was effective with single phage dose treatment belonged to *Podoviridae* having a life cycle with an adsorption velocity of 4.35 × 10^− 9^ mL/min, latent period of 40 min and a burst size of 120 phage particles/infected cell. *Enterobacter* phage ELP140 was found to have a shorter life cycle with an adsorption velocity of 2.8 × 10^− 9^, 11 min latency period with the release of 135 phages/infected cell, and *Escherichia* phage ECP311 having a life cycle with an adsorption velocity of 1.1 × 10^− 9^, 26 min latency period with the release of 110 phages/infected cell. The latter two phages ELP140 and ECP311 required more than a single dose for recovering larvae from bacterial infection.

The application of multiple phage doses to eliminate the bacteria (pathogen) from the larvae could also prove that the use of single phage (instead of cocktail of phages) should be sufficient to achieve the 100% survival rate when targeting the single bacterial infections. But earlier studies showed the use of cocktail of phages to eliminate the bacterial infection [21, 22]. This study shows that the effectiveness of phage therapy depends only of the ability of single phage to reduce the bacterial load and not on the complexity of phage preparations. This study also highlights the use of phage cocktails in eliminating the multiple bacterial infections which showed 100% survival rate of larvae with four doses of prepared phage cocktails. To the best of our knowledge, this is the first study to report the use of phage cocktails to cure multiple bacterial infections in *G. mellonella* larvae model. The data obtained from phage cocktail studies clearly showed that the phages can act synergistically to eliminate the bacterial infection. Our study reports the efficiency of phage cocktail against multiple/mixed bacterial infections. The studies using phage cocktails are complicated in their application because four/five continuous phage dose (at 6 h interval) are required to achieve 100% survival rate, so during therapy continuous monitoring of patient, immune complexity and effectiveness of patient should be carefully examined. Further, thorough investigation on phage cocktail will help in improving the clinical outcome of phage therapy and in treatment of patients infected with multiple pathogenic strains of bacteria. The route of administration of phages was found to have a profound effect on therapeutic outcome of the patients [18, 21, 23]. In some cases, a single phage dose treatment was not only found to be less effective but it also worsened the larval condition when tested for more than 72 h. The reason may be due to the rapid release of lipopolysaccharides (LPS) during the compound bacterial killing that causing deteriorated animal health [21, 24]. In order to avoid this during phage therapy, the use of single complex dose of phages should be avoided so that the side effects during bactericidal application and due to toxicity can be eliminated. The use of bacteriophage cocktail was found to effective in the treatment of *Acinetobacter baumannii* infection which saw the administration of bacteriophages intravenously and percutaneous into the abscess cavities, led to the reversal of the patient’s deteriorating clinical outcome, clearing all infection and return to health [25]. The treatment of Gram-negative bacterial infections is more complicated because the administration of any bacteriolytic agents can result in inflammatory responses (syndrome) due to toxic release; thus the choice of therapy/administration should be selected carefully.

## Conclusion

In this study, the route of administration of phages to the larvae was by intrahemocoelic (injection in to the last left pro-leg) which is comparable to the intravenous mode of administration in vertebrates. Considerably, the studied phages will have profound effect on vertebrates when they are used intravenously. Though our study showed some promising results for the studied phages using a simple animal model (wax moth larvae), a more detailed study is required to understand the dynamic relation of bacteriophages with the host bacterium and the various factors that govern this before any clinical trials are performed. In this study, we found that a single phage dose is enough to abolish the lethality (90% survival) of all the three bacterial infections in vivo, but to obtain 100% survival rate at least three phage doses are required at 6 h interval. For the first time, our study showed 100% survival of in vivo animal models when phage cocktail is used against mixed bacterial infections. This study had proven that a simple animal model like wax moth larvae is sufficient to evaluate the preliminary outcome of phage therapy. Future work should be considered using the phages against broader bacterial hosts and efficiency of treatment must be tested in more complex animal models.

## Methods

### Bacterial isolates and bacteriophages

A total of three multi-drug resistant Gram-negative bacteria *Escherichia coli* ec311, *Klebsiella pneumoniae* kp235 and *Enterobacter cloacae* el140 collected from diagnostic centers in Tamil Nadu, India were examined in this study. The bacterial identification was performed using VITEK identification system and 16S rRNA analysis. All three bacterial isolates were found to be resistant to ampicillin, cefotaxime, cefepime, gentamycin, amoxyclav, carbapenem and colistin. Bacteriophages were isolated from sewage treatment plants and initial screen showed that they had a broad host-range activity and were stable. The phages were named as *Escherichia* phage ECP311 (ECP311), *Klebsiella* phage KPP235 (KPP235) and *Enterobacter* phage ELP140 (ELP140). The invitro lytic activity data and one-step growth curves of the three phages are depicted in Additional file 2: Table S1 and Additional file 1: Figure S1 respectively.

### In vitro phage activity

For testing in vitro phage activity; spot test, phage plaque assay and broth propagation assay were performed as described earlier [17]. I) The bacterial lawn culture was prepared in Luria Bertani (LB) agar plate and 10 μL of respective phage lysate was spotted. The plates were incubated for 8 h and the appearance of bacterial clearance zone on the spots indicated the phage lytic activity. II) For phage plaque assay, 400 μL of bacterial culture (O.D_600_ = 0.65) was mixed with 200 μL of phage lysate and incubated at 37 °C for 10–15 min. The preparation was added to 3 mL of molten soft agar (0.75%) and mixed well and poured on solidified LB agar plates. The plates were allowed to solidify and incubated at 37 °C for 12 h and plaques were estimated. III) In the broth propagation assay, 50 μL of phage lysate was added to the 100 mL of bacterial inoculum. From the mixture, 100 μL was removed for every 2 h and 1:10 dilution was spread on LB agar plate. The plates were examined for reduction in bacterial count for 12 h at 2 h interval. Multiplicity of Infection (MOI) was calculated as the number of phage particles (PFU) divided by the number of bacterial cells (CFU). The stability of these phages was tested at different pH conditions by exposing the phages in the varying pH buffers ranging from 1 to 14 and at temperatures up to 70 °C.

### In vivo studies - G. Mellonella as an infection model

**Fig. 4:**
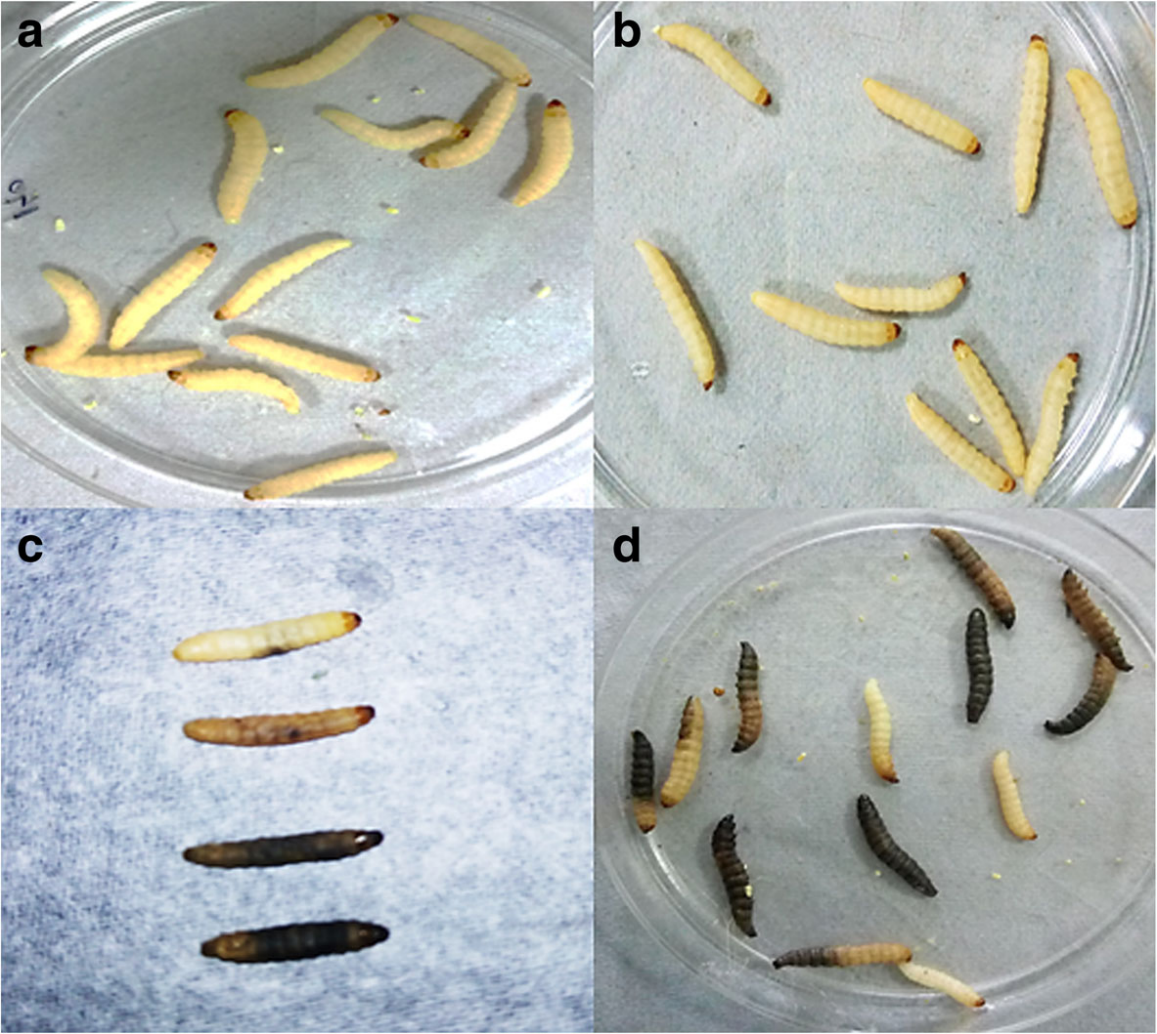
Morphology of *G. mellonella* used for the study, impact of phage treatment and the effect of bacterial infection. **a** Larva injected with saline (used as a control), **b** Larva survived after phage treatment, **c** Larva infected with bacteria, representation of different melanization observed on larvae, **d** Larva infected with bacteria alone (used as a control). The worms appearing black (melanization) or no movement were considered as dead and the worms that had movement without stimulation or no melanization were considered as alive

For in vivo studies, *Galleria mellonella* were used, the moths were obtained from Department of Entomology, University of Agricultural Sciences, Gandhi Krishi Vignana Kendra, Bengaluru, India. The moths were received at their young larval stage (app. 5 mm in length) and grown using artificial food (Corn meal: 400 g, Wheat flour: 200 g, Milk powder: 200 g, Yeast tablets: 100 g, Honey: 400 ml, Glycerin: 300 ml) at 35 °C in Antibiotic Resistance and Phage Therapy Laboratory, VIT, Vellore, India. Once the worms reached their late larval stage (app. 2–2.5 cm, creamy white color), they were used for evaluating the potential of bacteriophages (Abedon et al. 2017). After the study, *G. mellonella* health index scoring was performed using the larvae survival as categorized by either alive or dead. The larvae were considered as dead only when there was no 7movement or minimal movement on stimulation or larva appearing black (melanization) or > 3 black spots on brown larvae (Abedon et al. 2017), and considered alive only when there was movement without stimulation and no melanization (Fig. 4).

### Phage treatment: In vivo assays

**Fig. 5:**
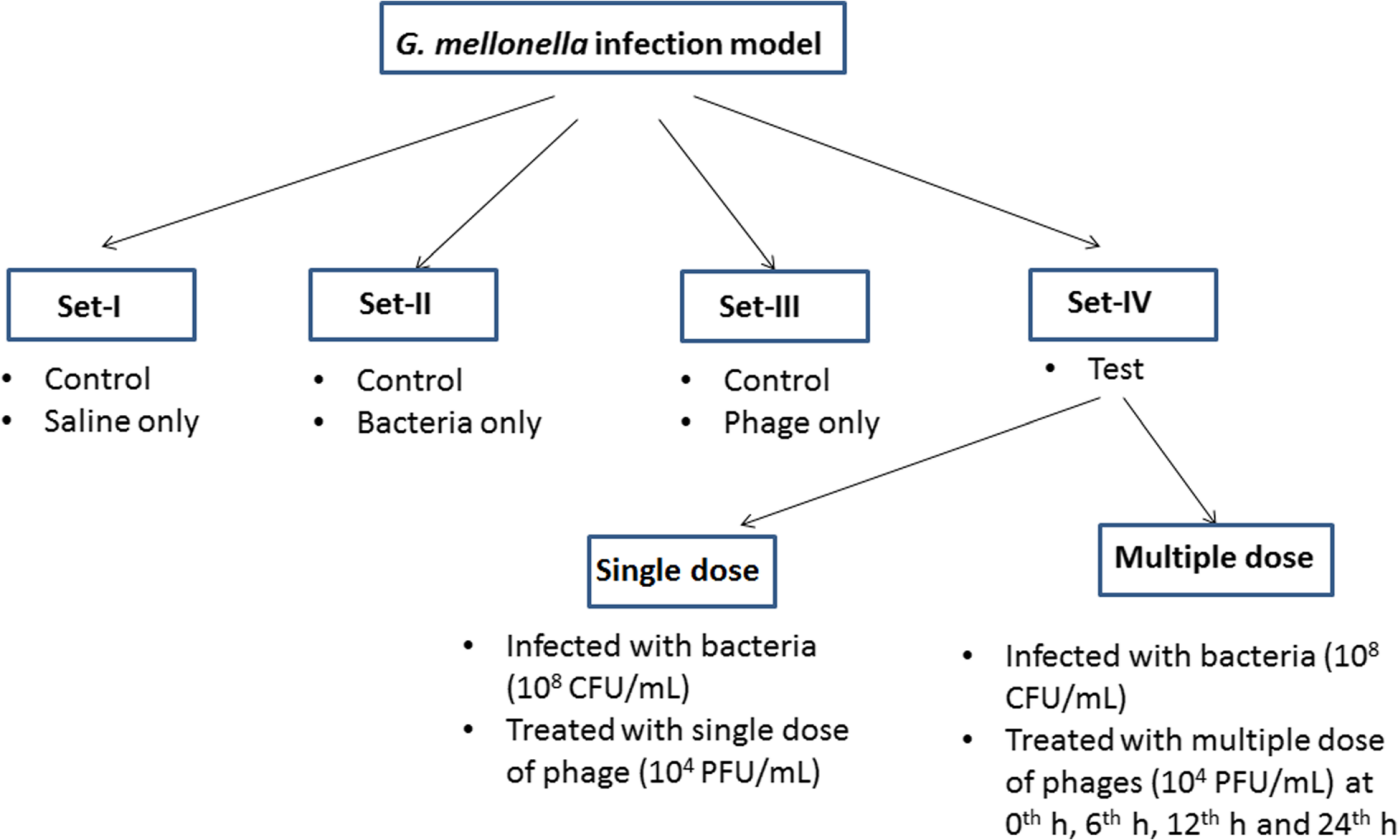
Diagrammatic representation of experiment setup used to evaluate the efficiency of phage therapy for the treatment of *G. mellonella* using single phage dose and multiple phage doses

Briefly, the host bacteria of *E. coli* ec311, *K. pneumoniae* kp235 and *E. cloacae* el140, were grown overnight and centrifuged at 8000 × g for 15 min. The resultant pellet was diluted to obtain the required 10^8^ CFU/mL (approx. O.D_600_ of 0.65) using LB broth and 20 μL of culture was used for infecting *G. mellonella*. The larvae were starved for 24 h before the experiment and both the bacterium and phage lysate was injected into the larva through the last left pro-leg. A set of 10 larvae per group was used. The study was conducted using three controls (Fig. 5); a) in set-1 all the larvae were injected with 20 μL of saline. b) in set-2 all the larvae were infected with 20 μL of bacterial inoculum and larval survivability was tested. c) in set-3 all the larvae were injected with 20 μL of prepared phage lysate and survivability was evaluated, d) set-4 (test), all the larvae were injected with bacterial inoculum (10^8^ CFU/mL) and treated with respective phage lysate (10^4^ PFU/mL). For assessing the antibacterial activity of phages in set-4, 20 μL of bacterial suspension was injected and within 1 h 20 μL of phage lysate was injected (for single phage dose). The larvae were tested for their survival at 12, 24, 48, 72 and 96 h, and the results were expressed as percentage survival. To determine the effect of multiple phage doses on larvae survival, four groups were maintained; once the larvae were infected with bacteria, the first phage dose was received at 0 h (group A-1 dose at 0 h), the second dose at 6 h (group B-2 doses at 0 h and 6 h), the third dose at 12 h (group C-3 doses at 0 h, 6 h and 12 h) and the fourth dose at 24 h (group D-4 doses at 0 h, 6 h, 12 h and 24 h). The treated larvae were evaluated for their survival at every 12 h up to 96 h and the bacterial enumeration from dead and live larvae was performed on LB agar by plating 0.1 mL of homogenized sample using the serial dilution method and the bacterial load of CFU per larva was enumerated. Five larvae from each group were examined and all the experiments were performed in triplicates and analysed using GraphPad Prism 7.0.

### Cocktail of phages against *G. mellonella* multiple bacterial infection

Larvae (*n* = 10) were infected with mixed bacterial culture (three bacteria at equal concentration each at 10^8^ CFU/mL) and treated using the phage cocktail (three phages at equal concentration each at 10^4^ PFU/mL). The prepared bacterial inoculum was mixed in the equal volume (100 μL each) and 20 μL from the mixed suspension was injected into the larvae. The infected larvae were treated using the prepared phage cocktail (20 μL) using a single dose (at 0 h), and with multiple doses; after 0 h (group-A- 1 dose), after 0 h and 6 h (group B- 2 doses), after 0 h, 6 h and 12 h (group C- 3 doses), after 0 h, 6 h, 12 h and 24 h (group-D 4 doses) and after 0 h, 6 h, 12 h, 24 h and 30 h (group E- 5 doses). The survivability was assessed at every 12 h up to 96 h and the results were interpreted as the percentage survival rates. All the experiments were performed in triplicates. The bacterial enumeration from dead and live larvae was performed on LB agar by plating 0.1 mL of homogenized sample using the serial dilution method and the bacterial load (CFU per larva) was enumerated. Five larvae from each group were examined and all the experiments were performed in triplicates and analysed using GraphPad Prism 7.0.

### Statistical analyses

The survival curves were plotted using Kaplan-Meier method and *log-rank test* was used to calculate the difference in survival rates using GraphPad Prism software 7.0 (GraphPad Software, Inc., La Jolla, USA). *p* < 0.05 was considered as statistically significant.

## Additional files

Additional file 1: **Figure S1**. One-step growth experiment results for the three phages: A) *E. coli* ec311, B) *K. pneumoniae* kp235, and C) *E. cloacae* el140. (DOCX 94 kb)

Additional file 2: **Table S1**. Host-range infection of the phages, *Escherichia* phage ECP311 (ECP311), *Klebsiella* phage KPP235 (KPP235) and *Enterobacter* phage ELP140 (ELP140). (DOCX 16 kb)

## Abbreviations

CFU: Colony forming units
ESKAPE: *Enterococcus faecium*, *Staphylococcus aureus*, *Klebsiella pneumoniae*, *Acinetobacter baumannii*, *Pseudomonas aeruginosa*, and *Enterobacter* species
MOI: Multiplicity of infection
PFU: Plaque forming units
