# Supplementary material for: The therapeutic potential of bacteriophages targeting gram-negative bacteria using Galleria mellonella infection model
Source: BMC Microbiol. 2018 Aug 31;18:97. doi: 10.1186/s12866-018-1234-4 (PMC6119258; doi:10.1186/s12866-018-1234-4)
Supplement: Supplementary file 1 — Figure S1. One-step growth experiment results for the three phages: A) E. coli ec311, B) K. pneumoniae kp235, and C) E. cloacae el140. (DOCX 94 kb) [file 12866_2018_1234_MOESM1_ESM.docx]

**Figure S1: One-step growth experiment results for the three phages: A) *E. coli* ec311, B) *K. pneumoniae* kp235, and C) *E. cloacae* el140.**

**
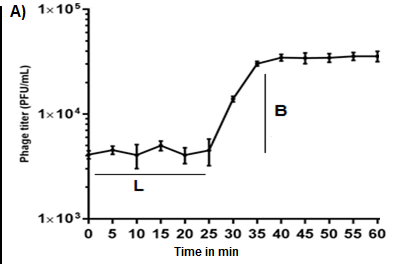
**

**
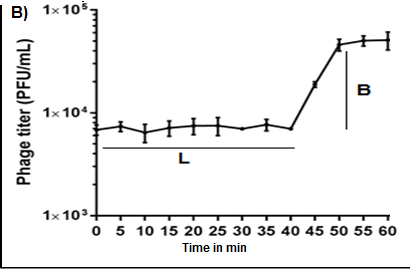
**

**
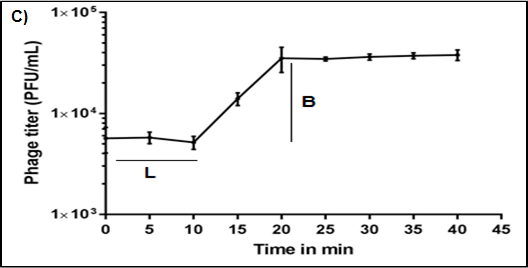
**

B-burst size; L-latent period
