# Supplementary material for: The therapeutic potential of bacteriophages targeting gram-negative bacteria using Galleria mellonella infection model
Source: BMC Microbiol. 2018 Aug 31;18:97. doi: 10.1186/s12866-018-1234-4 (PMC6119258; doi:10.1186/s12866-018-1234-4)
Supplement: Supplementary file 2 — Table S1. Host-range infection of the phages, Escherichia phage ECP311 (ECP311), Klebsiella phage KPP235 (KPP235) and Enterobacter phage ELP140 (ELP140). (DOCX 16 kb) [file 12866_2018_1234_MOESM2_ESM.docx]

**Table S1: Host-range infection of the phages, *Escherichia* phage ECP311 (ECP311), *Klebsiella* phage KPP235 (KPP235) and *Enterobacter* phage ELP140 (ELP140).**

| **Bacterial isolate** | **Pathotype/ serotype** | **Spot test** | **Agar-overlay** |
| --- | --- | --- | --- |
| *E. coli* EC1 | EPEC | + | +++ |
| *E. coli* EC2 | EPEC | + | + |
| *E. coli* EC3 | EPEC | + | ++ |
| *E. coli* EC4 | EPEC | + | + |
| *E. coli* EC5 | EPEC | + | ++ |
| *E. coli* EC6 | EPEC | + | + |
| *E. coli* EC7 | EPEC | + | + |
| *E. coli* EC8 | EPEC | + | +++ |
| *E. coli* EC9 | EPEC | + | + |
| *E. coli* EC10 | EPEC | + | + |
| *E. coli* EC11 | EPEC | + | + |
| *E. coli* EC12 | EPEC | + | +++ |
| *E. coli* EC13 | EHEC | + | + |
| *E. coli* EC14 | EHEC | + | + |
| *E. coli* EC15 | EHEC | + | +++ |
| *E. coli* EC16 | EHEC | + | ++ |
| *E. coli* EC17 | EHEC | + | + |
| *E. coli* EC18 | EHEC | + | + |
| *E. coli* EC19 | ETEC | + | + |
| *E. coli* EC20 | ETEC | + | +++ |
| *E. coli* EC21 | ETEC | + | +++ |
| *E. coli* EC22 | ETEC | + | ++ |
| *E. coli* EC23 | ETEC | + | + |
| *E. coli* EC24 | ETEC | + | + |
| *E. coli* EC25 | ETEC | + | + |
| *E. coli* EC26 | ETEC | + | +++ |
| *E. coli* EC27 | ETEC | + | +++ |
| *E. coli* EC28 | EIEC | + | +++ |
| *E. coli* EC29 | EIEC | + | + |
| *E. coli* EC30 | EIEC | + | +++ |
| *E. coli* EC31 | EIEC | + | + |
| *E. coli* EC32 | EIEC | + | ++ |
| *E. coli* EC33 | EIEC | + | + |
| *E. coli* EC34 | EIEC | + | +++ |
| *E. coli* EC35 | EAEC | + | ++ |
| *E. coli* EC36 | EAEC | + | +++ |
| *E. coli* EC37 | UPEC | + | + |
| *E. coli* EC38 | UPEC | + | ++ |
| *E. coli* EC39 | UPEC | + | + |
| *E. coli* EC40 | UPEC | + | +++ |
| *E. coli* EC41 | UPEC | + | ++ |
| *E. coli* EC42 | UPEC | + | +++ |
| *E. coli* EC43 | UPEC | + | +++ |
| *K. pneumoniae* KP1 | K1 | + | +++ |
| *K. pneumoniae* KP2 | K1 | + | + |
| *K. pneumoniae* KP3 | K1 | + | + |
| *K. pneumoniae* KP4 | K1 | + | +++ |
| *K. pneumoniae* KP5 | K2 | + | + |
| *K. pneumoniae* KP6 | K2 | + | +++ |
| *K. pneumoniae* KP7 | K5 | + | +++ |
| *K. pneumoniae* KP8 | K5 | + | +++ |
| *K. pneumoniae* KP9 | K5 | + | +++ |
| *K. pneumoniae* KP10 | Unknown | + | ++ |
| *K. pneumoniae* KP11 | Unknown | + | + |
| *K. pneumoniae* KP12 | Unknown | + | +++ |
| *K. pneumoniae* KP13 | Unknown | + | ++ |
| *K. pneumoniae* KP14 | Unknown | + | +++ |
| *K. pneumoniae* KP15 | Unknown | + | + |
| *K. pneumoniae* KP16 | Unknown | + | ++ |
| *K. pneumoniae* KP17 | Unknown | + | +++ |
| *E. cloacae* EL1 | - | + | +++ |
| *E. cloacae* EL2 | - | + | +++ |
| *E. cloacae* EL3 | - | + | +++ |
| *E. cloacae* EL4 | - | + | +++ |
| *E. cloacae* EL5 | - | + | +++ |
| *E. cloacae* EL6 | - | + | +++ |
| *E. cloacae* EL7 | - | + | +++ |
| *E. cloacae* EL8 | - | + | +++ |
| *E. cloacae* EL9 | - | + | +++ |
| *E. cloacae* EL10 | - | + | +++ |
| *E. cloacae* EL11 | - | + | +++ |
| *E. hormaechei* EH1 | - | + | + |
| *E. hormaechei* EH2 | - | + | + |
| *E. asburiae* EA1 | - | + | ++ |
| *E. asburiae* EA2 | - | + | ++ |
| *E. aerogenes* EG1 | - | + | + |
| *E. aerogenes* EG2 | - | + | + |

‘+’ - Positive in spot test and agar overlay results: ‘+++’ - 90% to 100% activity as observed in host bacterium, ‘++’ - 50% to 90% activity as observed in host bacterium, ‘+’ - <50% 90% activity as observed in host bacterium.
